# The Impact of the “Belt and Road Initiative” on International Scholarship Students

**DOI:** 10.3389/fsoc.2022.793018

**Published:** 2022-04-27

**Authors:** Lea Shih, Wei Cao

**Affiliations:** Department of Business Administration, Trier University, Trier, Germany

**Keywords:** China, scholarship students, difference-in-differences, education aid, Belt and Road Initiative (BRI), gravity model

## Abstract

The Belt and Road Initiative (BRI) has had a significant impact on China in political, economic, and cultural terms. This study focuses on the cultural domain, especially on scholarship students from the countries that signed bilateral cooperation agreements with China under the BRI. Using an integrated approach combining the difference-in-differences method and the gravity model, we explore the correlation between the BRI and the increasing number of international scholarship students funded by the Chinese government, as well as the determinants of students' decision to study in China. The panel data from 2010 to 2018 show that the launch of BRI has had a positive impact on the number of scholarship students from BRI countries. The number of scholarship recipients from non-BRI countries also increased, but at a much slower rate than those from BRI countries. The sole exception is the United States, which has trended downward for both state-funded and self-funded students.

## Introduction

Since its launch in 2013, China's Belt and Road Initiative (BRI) has received response from more than 100 countries, regions and international organizations. The core of the BRI is the networking of countries and people who not only trade with each other but also engage in cultural exchange, as cultural exchange is the basis on which transnational cooperation can be enhanced in all aspects (Luan and Sun, 2018; Kuah, 2019; Michael, 2020). Studying in China belongs to the essential part of cultural exchange within the framework of BRI. According to the latest release from the Chinese Ministry of Education (MOE), there were close to 500.000 international students from 196 countries and regions by 2018, ranking China as the third popular destination country for international students after the United States and the United Kingdom. It is remarkable that 77% of the international Students hail from Asia and Africa. In contrast, the number of students from North America has been shrinking since 2014, which was the only region with a declining trend (MOE, 2019). Since many countries in Asian and Africa are overlapping with the countries that signed bilateral cooperation agreements with China regarding BRI, we assume that similar structure can be found in the following analysis.

Beyond this quantitative growth, China has also undergone several structural changes in terms of the international student education by 2018. First, the number of **full degree students**, a critical indicator of the quality of higher education, increased significantly, especially the students from Africa countries grew the fastest (up 183% compared to 2013), while the scholarships students from Asian countries pursuing full degree studies in China expanded the fastest (up 111% compared to 2013). In addition, **engineering** has become the most popular major among the scholarship students, with an increase of 123.90% compared to 2013. For comparison, more than 50% of students from western countries such as United States, the United Kingdom, Germany, and France visited China on exchange and short-term programs to immerse themselves in the language and culture (Zhou and Wit, 2019). This difference indicates that China's attractiveness for international students varies greatly by country.

According to Zheng and Ma (2016), international students studying in China from the BRI countries had already steadily increased from 2004 to 2014, that was before the introduction of the BRI, however, their research provides very little evidence on scholarship students. We assumed this part was the most dependent on policy and the change in policy should be directly reflected in the figures, thus, we focused on this specific part of the international students. Since 2016, the Chinese Ministry of Education additionally added the Silk Road Scholarship to the original scholarship and offered 10,000 government scholarships to BRI countries each year (MOE, 2016). This measure was intended to encourage more students from BRI countries to study in China, which is considered the first evidence of the extent to which the BRI influences the decision of international scholarship students.

Our paper is organized as follows: the first section reviews the literature on push and pull factors affecting the mobility of international students, focusing primarily on Chinese scholarship policy related to the BRI, which is considered a crucial pull factor for scholarship students. The second section explains in detail the empirical methodology and data analysis we conducted to examine the conjunction of different pull and push factors that influence both international students and grant recipients. The third section summarizes the results of the empirical analysis, and the final section wraps up with a discussion.

## The Push and Pull Factors of International Student Mobility

There are widely varying factors affecting international student mobility. In many studies, these factors are classified into two categories: “Push” factors include those that operate within the country of origin to influence the decision of international students to study abroad. “Pull” factors include those factors that are found in the destination country and make it attractive to international students. Since the final decision of international students to choose a particular country is based on the conjunction of the push and pull factors in both the home and destination home countries. Explanations about the international student mobility therefore vary from country to country (Altbach, 1998; Mazzoarol and Soutar, 2002; Wei et al., 2018; Yuan and Gao, 2020).

Regarding China, the most available studies discuss the pull factors such as the economic development, geographical distance, quality of education, language similarity, acceptance of Chinese culture, cost issues (including the living expenses, travel costs etc.) and work opportunities after graduation etc. And the different effects of these factors on students from developing and developed countries has been noted in several studies. According to Song and Liu, students from developing countries are more attracted by the development prospect and relatively high education level in China compared to their origin countries, while students from developed countries are more likely to be affected by domestic happenings (Song and Liu, 2014). Moreover, international students from developing countries put the same weights on educational and economic factors for peer developing countries as potential destinations, while only economic factors are taken into consideration for developed countries as potential destinations (Liu et al., 2013; Wei, 2013). The effect of scholarship has only been marginally researched, according to Wei, government granting could to some extent offset the negative effects of distance and language differences to bring international students to China (Wei et al., 2018). However, the previous studies mostly did not distinguish government grant recipients from self-financed students. As scholarship students are an integral part of international students, do the above factors also influence the decision of the scholarship recipients in the same way? Or are they more influenced by the government policies of the destination countries that presumably reduce the effects of overall push factors in home countries? Before we examine the differential impact of pull and push factors in the empirical section below, we briefly review the evolution of China's policy on international students, which essentially frames the BRI and is considered an important pull factor for international scholarship students (see Table 1).

**Table 1:** China's policy on international students.

	**International students related policy**	**Major happenings**
1950	Started student exchange with former socialist countries in Eastern Europe	Establishment of diplomatic relations with socialist Bloc
1956	Started funding students from developing countries in Asia and Africa	China's Participation in Bandung Conference (1955), announcement of close cooperation with developing countries
1975	Started student exchanges with western countries	Visit of former U.S. President Nixon to China (1972) and resumption of diplomatic relations with Western countries
**1984**	**Education aid became a major component of China's foreign aid, along with economic aid**	**China's domestic policy shifted to economic reconstruction (1978)**
1989	Admission of international students was delegated to universities and opened to privately funded students from abroad	Decentralization of education administration by the Chinese central government (1985)
1996	Establishment of China Scholarship Council (CSC)	Large-scale reform of the higher education system in China (1995)
**2003**	**Launching the internationalization strategy in Chinese higher education, rapid growth in the number of international students**	**China's entrance into the WTO (2001)**
2010	Release of ”Study in China”—Program (2010–2020) by the MOE. The targets by 2020, China should become the largest destination country of international students in Asia	
2016	**Introduced Silk Road scholarship and offered BRI countries an additional 10,000 government scholarships per year**	**Announcement of BRI by state president Xi Jinping (2013)**

*Sources: MOE (2010, 2016), Ma and Zhao (2018), and Li (2020)*.

In retrospect, China started hosting the international students through student exchange, which usually followed the establishment of diplomatic relations with other countries and was government-oriented rather than university-oriented (see Table 1). In the 1950s, for example, it primarily served to support the independence movement of the former colonial states in Asia and Africa, following China's participation in the 1955 Bandung Conference, which promoted economic and cultural cooperation among developing countries. Then in the 1970s, students from Western countries were allowed to study in China after diplomatic relations between the U.S. and other Western countries and China normalized. However, the student exchange at that time was limited to a modest number due to a tight budget (Wen, 2018; Wu and Chan, 2019).

The first turning point occurred in 1984, when educational assistance was formally incorporated into China's foreign aid program together with economic cooperation. The Chinese government then began to gradually increase the number of government scholarships, which became the most frequently mentioned in official documents as an essential instrument of education aid to other developing countries (Yuan, 2014). This trend remained unchanged until the launch of the BRI, and China extended its scholarships to even more developing countries.

Another fundamental shift was the transformation of the Chinese education system in the 1990s. With the adoption of the “Higher Education Law,” the Chinese universities were assigned responsibility in the recruitment of international students, including the selection of scholarship applicants. Since then, universities have emerged as key actors in the recruitment, enrollment, management and teaching of international students. The number of international students jumped after that, and the number of self-funding students overtook government scholarship students to become the majority of international students (Li, 2020). After entering the WTO in 2001, Chinese universities pursued an internationalization strategy primarily aimed at enhancing their reputation internationally as providers of educational assistance. In this context, the Chinese Ministry of Education began to adopt several long-term programs for attracting international students. The most far-reaching program was called “Study in China” which ran from 2010 to 2020. The main targets of this program were to host 500,000 international students by 2020 and make China the largest destination country for study abroad in Asia. It also proposed a steady increase in Chinese government scholarships for international students, but without explicitly mentioning country preference (MOE, 2010). In retrospect, the main target of “Study in China” was according to statistics published by the Chinese Ministry of Education already achieved in 2018 (MOE, 2019).

The most recent boost for recruiting international students was the implementation of the BRI. Since 2013 the Chinese President Xi Jinping has announced several times at Forum on China-Africa Cooperation or during his visits to Indonesia and South America, etc., to increase government scholarships to developing countries. Furthermore, the Chinese Ministry of Education announced in 2015 an additional 10,000 government scholarships named “Silk Road Scholarship” would be offered annually to the student from BRI countries (MOE, 2016). Some provincial governments also adopted policies to support international students, often specifying specific geographic regions, such as Yunnan, a border province in southern China, which focuses on Southeast and South Asian countries, Guangxi, which focuses on ASEAN countries, and Inner Mongolia in northern China, which has scholarships for international students from Mongolia. As of 2018, a total of 30 provincial governments that have set up scholarship programs for international students (Lu, 2021). Under the BRI, various forces worked together to attract international students coming to China. The extent to which they are effective, however, depends on their conjunction with other factors, which we explore in more detail in the following section.

## Data Analysis

### Difference-in-Differences Method

To examine whether the change in the number of grantees from BRI countries is related to the implementation of the BRI, we will use the difference-in-differences (DID) method, which provides a more precise picture of the differential effects before and after the implementation of the BRI, while controlling for group and time effects. The basic principle of the DID model is to group the samples twice. The first differentiation before and after the launch of the BRI generally only reflects the apparent effect. Because additional determinants besides the BRI before and after the launch of BRI in 2014 also differed, the effect of the policy implementation cannot be determined by comparing the difference in the number of scholarship students alone, the different trends between the treatment group and the control group should be additionally examined.

In the following, we will divide the data of scholarship students into two groups according to their home countries. The treatment group includes the countries that have signed bilateral cooperation agreement with China regarding BRI, while the control group include those countries that did not signed official cooperation agreements. As we only have data of scholarship recipients through 2018, those countries that signed cooperation agreements after 2018 are assigned to the control group rather than the treatment group. Based on the data collected before and after 2014, the amount of change in the dependent variable for the treatment group can be calculated. For the control group, the change in the same variable was also calculated before and after 2014. Finally, the difference between the two variables is calculated, which is a common method for estimating the treatment effect.

The basic regression model can be based on the generalized linear model expression for DID therefore be obtained as follows:


(1) $$\begin{array}{l} \ln ScholarshipStudent_{jt}=\beta_{0}+\beta_{1}treat_{j}+\beta_{2}post_{t}+\\ \;\beta_{3}treat_{j}\cdot post_{t}+\varphi X_{ijt}+\varepsilon_{ijt} \end{array}$$

where j and t represent country and time, respectively. The dependent variable, ln *ScholarshipStudent*_*jt*_, represents the number of students from country j who received a scholarship in year t; treat_j_ denotes a country fixed effect and post_t_ denotes a year fixed effect. The core explanatory variable treat_j_·post_t_ indicates whether country j signed up for the BRI in year t. The coefficient β_3_ indicates the growth effect of the change in the number of scholarship students after the BRI was carried out. ϕX_ijt_ indicates other observable control variables and ε_ijt_ indicates the error term.

Furthermore, we conducted a robustness check by changing the year in which the BRI was introduced to exclude the possibility that it may have affected common trends between the treated and untreated groups. This allows us to check whether the number of scholarship holders was also increased in another year.

### Gravity Model

The gravity model was originally applied to measure the determinants of international trade flows. In recent years, it has been used extensively in the study of migration, tourism, international student mobility, and other topics (Beine et al., 2014; Song and Liu, 2014). In this study we combine gravity model with DID method to obtain a more accurate econometric model. In the Model 1, ϕX_ijt_ denotes the control variables, which include both proxies for the core explanatory variables as well as dummy variables. In this study, we consider the common determinants such as GDP per capita, trade flow, geographical distance, and common language tested in the Gravity model as the control variables denoted with ϕXijt. Using a logarithmic form, the model 1 is rewritten in the following form after the inclusion of control variables:


(2) $$\begin{array}{l} \ln ScholarshipStudent_{jt}=\beta_{0}+\beta_{1}treat_{j}\cdot post_{t}+\beta_{2}\ln GDP_{jt}+\\ \;\beta_{3}\ln dist_{ij}+\beta_{4}\ln Tradeflow_{ij}+\beta_{5}D_{Chinese}+\varepsilon_{ijt} \end{array}$$

where the interaction term *treat*_*j*_·*post*_*t*_ constructed in this way represents whether country j was involved in BRI in year t, which is the core explanatory variable in this section. ln *GDP*_*jt*_ denotes the gross domestic product of the scholarship student's country of origin j; ln *dist*_*ij*_ denotes the geographical distance from scholarship student's country of origin capital j to China's capital I; *lnTradeflow*_*ij*_ denotes the **b**uying and selling of goods and services between China i and country of origin j; the dummy variable _*D*_*Chinese*_*j*_ indicates whether Chinese is used in the official and private sectors of the country of origin.

### Sample Selecting

The variables are defined in Table 2. The dependent and control variables are in logarithmic form, except for the dummy variables.

**Table 2:** Variable description.

**Study variables**	**Variable representation**	**Definition and data source**
Explained variables	*Scholarship* *Student_*jt*_*	Number of students receiving Chinese Government Scholarships, using “Brief Statistics of International Students in China” data
Core explanatory variables	*treat_*j*_·post_*t*_*	Dummy variable indicates the occurrence of the BRI. This variable indicates whether country j is a BRI country in period t
Control variables	*GDP_*jt*_*	Gross Domestic Product of country j in period t, World Bank
	*Tradeflow_*ij*_*	Trade flow as reported by the exporter (in thousands current US$), data from Comtrade
	*dist_*ij*_*	Geographical distance between Beijing and the capitals of country j, using GeoDist data from CEPII
	*D_*chinese*_*	Dummy variable indicates whether Chinese is used in the official and private sectors of the country j, using GeoDist data from CEPII

### Dependent Variable

The sample of the dependent variable, ln *ScholarshipStudent*_*jt*_ selected for this section is based on Brief Statistics for International Students in China, published by the Ministry of Education of China. We collected panel data from 2010 to 2018 on countries and regions with or more than 500 international students coming to China, covering 108 countries and regions in total, and including both self-funded and scholarship students. Among them, the statistics of students from South Sudan coming to China in 2010–2011 are missing because the Republic of South Sudan became independent from Sudan in 2011, so the sample data of South Sudan was excluded from the data collection and regression analysis. The sample therefore comprises a total of 107 countries and regions which have a total number of students coming to China of more than 500.

We divide these 107 countries into treatment and control groups (see Table 3). The treatment group contains 38 countries that signed cooperation agreements with China as of 2018. Some countries such as Bosnia and Herzegovina, Greece, Azerbaijan, and Lebanon have also signed agreements, but from which <500 students had come to China and no data are available, they are not included in this study. The remaining 68 countries then form the control group. The ranking in the following table is in descending order of the number of scholarship students.

**Table 3:** Samples of treatment group and control group.

**Group**	**Countries and regions**
Treatment group (*N* = 38)	South Korea, Thailand, Russia, Pakistan, Indonesia, Vietnam, Kazakhstan, Mongolia, Laos, Malaysia, Singapore, Nepal, Myanmar, Bangladesh, Philippines, Kyrgyzstan, Uzbekistan, Ukraine, Tajikistan, Turkmenistan, Turkey, Sri Lanka, Cambodia, Saudi Arabia, Poland, Iran, Egypt, Jordan, Belarus, Syria, Portugal, Iraq, Afghanistan, Austria, Bahrain, Czech Republic, Romania, Hungary
Control group (*N* = 69)	United States, Japan, India, France, Germany, United Kingdom, Italy, Australia, Ghana, Canada, Nigeria, Yemen, Tanzania, Spain, Ethiopia, Zambia, Zimbabwe, Netherlands, Sudan, Cameroon, Mexico, Kenya, South Africa, North Korea, Sweden, Brazil, Congo (RC), Belgium, Somalia, Congo (DRC), Rwanda, Denmark, Mauritius, Morocco, Uganda, Colombia, Switzerland, Madagascar, Burundi, new Zealand, Algeria, Guinea, Norway, Equatorial Guinea, Finland, Niger, Mali, Angola, Ivory Coast, Senegal, Ireland, Sierra Leone, Benin, Namibia, Peru, Venezuela, Panama, Chile, Djibouti, Liberia, Mozambique, Malawi, Ecuador, Botswana, Argentina, Tunisia, Gabon, Papua New Guinea, Jamaica

### Control Variables

*GDP*_*jt*_: Size of the economies of the countries of origin of the scholarship students. GDP per capita is the main indicator of a region's development. The higher the GDP of a region, the higher the level of economic development and the more frequent the economic activity.

*Tradeflow*_*ij*_: The volume of trade between the countries of origin of the scholarship students and China. The higher the frequency of bilateral trade, the higher the exchange of economically active people between the two countries.

*dist*_*ij*_: The geographical distance between the scholarship student's country of origin and the country of destination. Geographical distance is also an important factor influencing international student mobility. Social, economic and cultural exchanges between two countries are easier if they are geographically close. The cultures between the two countries are likely to be more similar, so the geographical distance variable can also represent the cultural factors variable to some extent. In contrast, the greater the geographical distance, the greater the cultural differences between the two countries, which usually has a negative impact on the transport costs as well as the psychological costs of going abroad (Beine et al., 2014; Liu et al., 2018).

*D*_*Chinesej*_: Dummy variables. It indicates whether the country of the student receiving the scholarship uses Chinese in the official and folk languages. The cultural differences between countries can be an obstacle to the cultural identification of international students with the destination country.

Table 4 shows the statistical descriptions of each control variable. For each of these variables, we collected 963 observations. It can be seen that the difference between the minimum, maximum, and standard deviation values of the number of scholarship students and the volume of trade flow is relatively large. It indicate that the distribution of scholarship students by country is uneven and that there are large differences in the volume of trade between their countries of origin and China.

**Table 4:** Descriptive statistics (including dummy variable).

**Variable**	**Observation**	**Minimum**	**Maximum**	**Mean**	**Std. Deviation**
*LnScholarshipStudent_*jt*_*	963	1.79	9.01	5.1143	1.15872
*lnGDP_*jt*_*	963	22.5	23.35	22.99	0.25428
*lndist_*ij*_*	963	6.70	9.87	8.9067	0.58644
*lnTradeflow_*ij*_*	963	10.77	20.15	15.6355	1.77458
*D_*chinese*_*	963	0	1	0.1214	0.32687

### Empirical Test

The Table 5 summarizes our test results. The *R*^2^ and *p*-values indicate that the overall model is statistically significant and that the model setup and choice of control variables are relatively reasonable. The results of the ordinary least squares (OLS) regressions with and without control variables show that the coefficient on the interaction term for *treat*_*j*_·*post*_*t*_ is significant and positive, indicating that the BRI is statistically correlated with the number of scholarship students. The coefficient (0.611, significant at the 1% level, without any control variables) shows that the number of recipients of national scholarships benefits from the BRI.

**Table 5:** Estimated results.

	**(1) OLS**	**(2) OLS**	**(3) FE**	**(4) FE**
treat_j_·post_t_	0.611*** (0.043)	0.236** (0.124)	0.611*** (0.057)	0.237** (0.133)
*lnGDP_*jt*_*		0.927*** (0.139)		0.931*** (0.197)
*lndist_*ij*_*		−1.138*** (0.071)		−0.917*** (0.072)
*lnTradeflow_*ij*_*		0.014 (0.019)		0.014 (0.024)
*D_*chinese*_*		0.057 (0.158)		0.057 (0.114)
Constant	4.891	8.253	4.735	8.356
Observations	963	963	963	963
*R^2^*	0.099	0.293	0.159	0.317
*P*-Value	0.103	0.011	0.000	0.066
Year fixed effects	No	No	Yes	Yes
Country fixed effects	Yes	Yes	Yes	Yes

*Values in brackets are standard deviations. The dependent variable of all models is number of students receiving Chinese Government Scholarships. Columns (1) and (2) present the OLS regression results with and without control variables, respectively. Columns (3) and (4) present the results of the fixed effects model with and without control variables, respectively*.

***p < 0.01; **p < 0.05.

For the above variables we ran ordinary least squares (OLS) as well as fixed effects regressions (FE), respectively. In Columns (l) and (2) we fixed the countries and could see that the R^2^ values were not ideal, with *R*^2^ values of 0.099 and 0.293, respectively, indicating that the model didn't fit well-enough to explain how the number of scholarship students was affected by the BRI. We therefore ran a further fixed effects regression. By running the regression with both the year and country factors fixed, we found that the *R*^2^-value in Column (4) outperformed that in Column (2) and the *p*-value was significant at the 10% level. Therefore, Column (4) was finally chosen for subsequent interpretation in this study.

As reported in the regression results, the coefficients on the variables *lnGDP*_*jt*_, *lnTradeflow*_*ij*_, and *D*_*chinese*_ were positive in all regressions, with GDP being significant at the 1% level of significance, which indicates a significant positive correlation between GDP and the total number of scholarship students coming to China. And the number of scholarship students from high-GDP countries is much higher compared to the number of scholarship students from low-GDP countries. Although international students from low-income countries are more sensitive to scholarships (Zong and Li, 2020), we find that the GDP value of the country of origin of the scholarship students also positively influences the number of scholarship recipients.

The results for the variable *lnTradeflow*_*ij*_as well as *D*_*chinese*_ were not significant. There is no significant relation between the number of students receiving scholarships and the trade between the two countries. And there is no significant correlation between the use of Chinese and the number of scholarship students. However, overall, Chinese is not spoken in a wide range of countries and regions. A large proportion of scholarship students also choose to study in China for the purpose of learning Chinese and learning about traditional Chinese culture, usually as short-term students. The common language does not greatly influence the international scholarship students in choosing the study destination.

We conducted a robustness test based on determining that the coefficient on the interaction term of *treat*_*j*_·*post*_*t*_ was significant and positive. The test was conducted by changing the year in which the policy was introduced. The introduction of the BRI is set at a period prior to 2014 to examine whether there is still a boosting effect on the number of scholarship students. Double differencing presupposes that there is no significant difference in the trend of growth in numbers between the treatment and control groups prior to the 2014. Therefore, if the policy event is set at a period prior to 2014 then the estimated coefficients on the core variables will be insignificant. If the results are contrary to expectations, then this implies that there are indeed some underlying unobservable factors that also drive the increase in the number of people in the treatment group, and that the boosting effect of BRI implementation is not the sole driving force. Therefore, three experiments were conducted with the timing of 2011, 2012, and 2013, respectively. The results of the tests are shown in Table 6. As can be seen from the test results, the estimated coefficients on the core variables of the interaction term are not significant and therefore the effect of other potentially unobservable factors on the number of scholarship students in this section can be excluded.

**Table 6:** Result of robustness test: change of time.

**Year**	**2011**	**2012**	**2013**
*treat_*j*_·post_*t*_*	0.259 (0.181)	−0.061 (0.109)	0.246 (0.151)
*p*-value	0.154	0.573	0.103

*Values in brackets are standard deviations*.

From the Robustness test it is possible to demonstrate in reverse that the BRI is statistically correlated with the number of scholarship students. On this basis we include control variables that have a measurable effect on the treatment effect, and the coefficient remains significant and positive after considering the control variables. Based on the OLS regression, we used a fixed effects model to estimate the treatment effect of the BRI and found that the coefficient was still positive and significant at the 5% level. It not only indicates that the BRI contributed to the increase in the number of scholarships, but also statistically verifying that the BRI did contribute to the number of scholarships from the 38 BRI countries selected for the sample. That is, the number of scholarship students from BRI countries is significantly different from the number of scholarship students from non-BRI countries.

## A Discussion of the Findings

This paper mainly explores the impact of the BRI on China from education perspective. Our empirical analysis combine DID method with gravity model and found that the number of scholarship students from countries along the Belt and Road is significantly higher than the number of scholarship students from non-BRI countries (significance level of effect of 1%). And among the factors affecting the number of scholarship students, geographical distance is negatively related to the number of scholarship students. In general, the greater the bilateral geographical distance, the higher the migration cost which weaken the propensity of international students to study abroad. However, this determent does not affect the scholarship students from the BRI countries, even though they are largely developing countries. Our results also validate (Wei and Lai, 2017) findings that students from developing countries tend to choose countries that are geographically farther away as study destinations. The GDP volume of the country of origin of the scholarship students is positively and significantly related to the number of scholarship students. It shows that the level of economic development between China and the country of origin of the scholarship students is an important economic factor contributing to the inflow of scholarship students. There is no significant effect of the volume of trade flow between the scholarship student's country of origin and China. In general, economic and trade exchanges can reduce the information cost of international students moving between two countries, and the larger the total amount of import and export commodity trade between two countries, the more favorable the inflow of international students to China (Wei et al., 2018). However, we did not confirm a significant effect of bilateral trade volumes on the number of scholarship students. However, in terms of the coefficient (0.014), Trade flow still has a positive effect on the number of scholarship students. The use of Chinese in the home countries has no significant impact on the number of scholarship students. The higher the linguistic similarity between the two countries, the higher their cultural similarity. However, in the case of scholarship students, linguistic similarity did not have a significant effect on them. The reason for this may be that there are very few countries in the world where Chinese is an official language. In addition, scholarship students choose Chinese as their major, and many of them come to China to study for the purpose of learning Chinese. Overall, the value of GDP as an economic factor and geographical distance as a socio-cultural factor, they have a higher impact on scholarship students in BRI countries than in non-BRI countries.

## Conclusion

The empirical research of this paper shows that the implementation of BRI has a boosting effect on the number of scholarship students. After 2014, the number of scholarship students from the BRI is significantly higher than the number of scholarship students from non-BRI countries. This finding suggest the Government policy of host country plays a crucial role in upgrading the supply side of higher education. Over time, this “pull” factor could likely reduce the impact of traditional “push” factors, especially with the increasing international competition to attract foreign students. Host governments and their education institutions need to consider the importance of “pull” factors that influence students' study destination choice. However, this study is limited by the fact that the BRI countries are not further subdivided by economic development level. Future research could be conducted to explore whether there is a difference in the BRI countries by further refining the groupings. It is also instructive for future research to examine the structural changes in the scholarship students, which will provide additional evidence on the quality of Chinese higher education and the effect of the Chinese education policy.

## Data Availability Statement

The original contributions presented in the study are included in the article/supplementary material, further inquiries can be directed to the corresponding author.

## Author Contributions

All authors listed have made a substantial, direct, and intellectual contribution to the work and approved it for publication.

## Funding

This study is funded under the Research Initiative of the Ministry of Science and Health Rhineland-Palatinate, Germany.

## Conflict of Interest

The authors declare that the research was conducted in the absence of any commercial or financial relationships that could be construed as a potential conflict of interest.

## Publisher's Note

All claims expressed in this article are solely those of the authors and do not necessarily represent those of their affiliated organizations, or those of the publisher, the editors and the reviewers. Any product that may be evaluated in this article, or claim that may be made by its manufacturer, is not guaranteed or endorsed by the publisher.

## Databases

GeoDist. *Research and Expertise on the World Economy*. Available online at: http://www.cepii.fr/cepii/en/bdd_modele/presentation.asp?id=6 (accessed February 10, 2021).

World Data. *Chinese Speaking Countries, Languages*. Available online at: https://www.worlddata.info/languages/chinese.php (accessed August 3, 2021).

World Bank Data. “*GDP,” Indicator*. Available online at: https://data.worldbank.org/indicator/NY.GDP.MKTP.CD?end=2018&name_desc=false&start=2010 (accessed February 12, 2021).

*Brief Statistics on International Students 2010*. Available online at: http://www.moe.gov.cn/jyb_xwfb/gzdt_gzdt/s5987/201103/t20110302_128437.html (accessed June 5, 2021).

*Brief Statistics on International Students 2011*. Available online at: http://www.moe.gov.cn/jyb_xwfb/gzdt_gzdt/s5987/201202/t20120228_131117.html (accessed June 5, 2021).

*Brief Statistics on International Students 2012*. Available online at: http://www.moe.gov.cn/jyb_xwfb/gzdt_gzdt/s5987/201303/t20130307_148379.html (accessed June 5, 2021). *Brief Statistics on International Students 2013*. Available online at: http://www.moe.gov.cn/jyb_xwfb/gzdt_gzdt/s5987/201402/t20140221_164235.html (accessed June 5, 2021).

*Brief Statistics on International Students 2014*. Available online at: http://www.moe.gov.cn/jyb_xwfb/gzdt_gzdt/s5987/201503/t20150318_186395.html (accessed June 5, 2021).

*Brief Statistics on International Students 2015*. Available online at: http://www.moe.gov.cn/jyb_xwfb/gzdt_gzdt/s5987/201604/t20160414_238263.html (accessed June 5, 2021).

*Brief Statistics on International Students 2016*. Available online at: http://www.moe.gov.cn/jyb_xwfb/xw_fbh/moe_2069/xwfbh_2017n/xwfb_170301/170301_sjtj/201703/t20170301_297677.html (accessed June 5, 2021).

*Brief Statistics on International Students 2017*. Available online at: http://www.moe.gov.cn/jyb_xwfb/gzdt_gzdt/s5987/201803/t20180329_331772.html (accessed June 5, 2021).

*Brief Statistics on International Students 2018*. Available online at: http://www.moe.gov.cn/jyb_xwfb/gzdt_gzdt/s5987/201904/t20190412_377692.html (accessed June 5, 2021).
